# Modelling the consequences of targeted selective treatment strategies on performance and emergence of anthelmintic resistance amongst grazing calves

**DOI:** 10.1016/j.ijpddr.2016.11.002

**Published:** 2016-11-16

**Authors:** Zoe Berk, Yan C.S.M. Laurenson, Andrew B. Forbes, Ilias Kyriazakis

**Affiliations:** a School of Agriculture Food and Rural Development, Newcastle University, Newcastle Upon Tyne, NE1 7RU, UK; b Animal Science, School of Environmental and Rural Science, University of New England, Armidale, New South Wales, 2351, Australia; c Scottish Centre for Production Animal Health and Food Safety, School of Veterinary Medicine, University of Glasgow, G61 1QH, Scotland, UK

**Keywords:** Cattle, Gastrointestinal parasitism, *Ostertagia ostertagi*, Targeted selective treatment (TST), Anthelmintic resistance, Mathematical modelling

## Abstract

The development of anthelmintic resistance by helminths can be slowed by maintaining refugia on pasture or in untreated hosts. Targeted selective treatments (TST) may achieve this through the treatment only of individuals that would benefit most from anthelmintic, according to certain criteria. However TST consequences on cattle are uncertain, mainly due to difficulties of comparison between alternative strategies. We developed a mathematical model to compare: 1) the most ‘beneficial’ indicator for treatment selection and 2) the method of selection of calves exposed to *Ostertagia ostertagi*, i.e. treating a fixed percentage of the population with the lowest (or highest) indicator values versus treating individuals who exceed (or are below) a given indicator threshold. The indicators evaluated were average daily gain (ADG), faecal egg counts (FEC), plasma pepsinogen, combined FEC and plasma pepsinogen, versus random selection of individuals. Treatment success was assessed in terms of benefit per R (BPR), the ratio of average benefit in weight gain to change in frequency of resistance alleles R (relative to an untreated population). The optimal indicator in terms of BPR for fixed percentages of calves treated was plasma pepsinogen and the worst ADG; in the latter case treatment was applied to some individuals who were not in need of treatment. The reverse was found when calves were treated according to threshold criteria, with ADG being the best target indicator for treatment. This was also the most beneficial strategy overall, with a significantly higher BPR value than any other strategy, but its degree of success depended on the chosen threshold of the indicator. The study shows strong support for TST, with all strategies showing improvements on calves treated selectively, compared with whole-herd treatment at 3, 8, 13 weeks post-turnout. The developed model appeared capable of assessing the consequences of other TST strategies on calf populations.

## Highlights

• Different phenotypic traits were evaluated for use in targeted selective treatments.
• Different methods of selection for treatment were evaluated.
• Benefit ratio was assessed as weight gain/frequency of resistant alleles in helminths.
• Benefit ratio declined as a larger percentage of the herd was treated.
• Treatment according to threshold triggers of average daily gain was most beneficial.

## Graphical abstract

**Figure:**
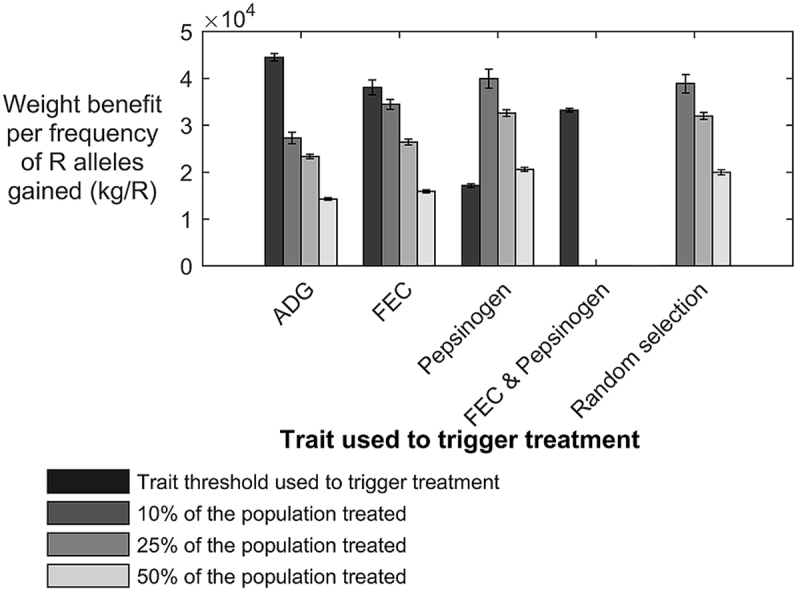

## 1 Introduction

The control of gastrointestinal parasitism for small ruminants has long been under threat from the development of anthelmintic resistance by parasite populations (Kaplan, 2004, Wolstenholme et al., 2004, Jabbar et al., 2006, Papadopoulos et al., 2012). However, in recent years it has become evident that this is also an emerging problem for cattle (Edmonds et al., 2010, Sutherland and Leathwick, 2011, O'Shaughnessy et al., 2014b, Rose et al., 2015). With nematode resistance now present to all three of the broad spectrum anthelmintic classes (benzimidazoles, levamisole and macrocyclic lactones) used on cattle (Sutherland and Leathwick, 2011), control strategies aiming to sustain effective parasitic control are of key importance.

Methodologies designed to maintain *refugia* within nematode populations can help to reduce the build-up of resistance by preserving susceptible nematode genotypes. A reservoir of susceptible genotypes on pasture helps to dilute the frequency of resistance alleles amongst nematodes and maintain anthelmintic efficacy (van Wyk, 2001, Gaba et al., 2010). One strategy that aims to achieve this is targeted selective treatment (TST), which involves the treatment of selected individuals that require, or will benefit from, treatment, as opposed to treatment of the entire group (van Wyk et al., 2006). Individuals are generally identified as needing to receive treatment on the basis of their level of parasitism or performance (Charlier et al., 2014). Although TST strategies have been developed and applied successfully in lambs (Greer et al., 2009, Kenyon et al., 2009, Kenyon et al., 2013), there are considerably fewer studies on cattle, with the first insights into the application of TST having occurred relatively recently (Greer et al., 2010, McAnulty et al., 2011, Höglund et al., 2013, O'Shaughnessy et al., 2014a, O'Shaughnessy et al., 2015a, O'Shaughnessy et al., 2015b). As there are important differences in host-parasite interactions and parasite epidemiology between cattle and sheep, differences in the methodology and application of TST in cattle can be expected.

Although TST strategies in sheep have been shown to be beneficial in reducing selection for anthelmintic resistance (Kenyon et al., 2013), it is difficult to know which of the various strategies would be most effective under various scenarios. At present there are no direct comparisons of TST strategies in cattle, in part due to difficulties arising from confounding variables (Höglund et al., 2013, O'Shaughnessy et al., 2015a). Additionally, it is difficult and time consuming to test such strategies in the long-term. Simulation modelling on the other hand may offer an effective alternative, and be highly beneficial in assessing the feasibility of novel control strategies. Here we address these gaps, by developing and using a simulation model that represents calf - *Ostertagia ostertagi* interactions and the epidemiology of the infection (Berk et al., 2016a, Berk et al., 2016b), in order to test the effectiveness of different TST approaches. *O. ostertagi* is the parasite of greatest significance in cattle grazing in temperate climates, and as the developed model is stochastic, it allows us to make predictions for the application of TST in a population of calves.

## 2 Materials and methods

The current model was based on the simulation approach of Berk et al., 2016a, Berk et al., 2016b, which aims to predict the effects of parasitism with *O. ostertagi* on a population of growing calves, taking into account host phenotype, host-parasite interactions and parasite epidemiology. The model has been further developed here to account for anthelmintic resistance amongst nematodes, by considering the susceptibility of each nematode genotype to anthelmintic treatment.

### 2.1 Host-parasite interactions

Briefly, it was assumed that a healthy calf attempts to ingest sufficient nutrient resources to meet demands for growth and maintenance (Coop and Kyriazakis, 1999). In the presence of parasitism, resource requirements increase due to endogenous protein losses to the calf (Fox, 1993). It is further assumed that the calf acquires immunity to reduce the impact of infection (Claerebout and Vercruysse, 2000), and by doing so further increases resource (e.g. protein) requirements. In addition to the endogenous protein loss and the increased resource requirements, a reduction in appetite and feed intake accompanies infection (Fox et al., 1989, Forbes et al., 2000, Kyriazakis, 2014). Although complex, the mechanism for inappetance in ostertagiosis was modelled as a function of the rate of immune acquisition, as it has been suggested that this reduction is associated with components of the immune response (e.g. cytokines), and related pathological and inflammatory responses (Fox et al., 1989, Kyriazakis, 2010, Kyriazakis, 2014). Consequently, the calf consumes insufficient resources to fulfil its requirements. Ingested protein is usually the first limiting nutrient resource. Once the protein loss due to parasitism has been accounted for it was assumed that allocation of limited resources were prioritised towards maintenance and repair (Coop and Kyriazakis, 1999). Remaining resources were then allocated between growth and immunity, proportional to their requirements (Kahn et al., 2000, Doeschl-Wilson et al., 2008, Laurenson et al., 2011). The model was parameterised such that the calf and its growth represented a weaned, castrated male (steer) Limousin x Holstein Friesian born in autumn (Berk et al., 2016a).

The individual calf model was extended to a stochastic model by considering between-animal variation in calf characteristics (Berk et al., 2016b); between-animal variation was assumed in intrinsic growth rate, body composition (expected protein and lipid content at maturity), maintenance requirements (protein and energy), and immune response traits (rate of acquisition, as well as initial and final rates for the immune traits of establishment, mortality and fecundity). The rates of acquisition in the three immune traits were assumed to follow a log-normal distribution, whereas all other traits were assumed to be normally distributed (Vagenas et al., 2007, Laurenson et al., 2012). Additionally the rates of immune acquisition for all 3 immune traits were assumed to be a function of overlapping effector mechanisms (Mihi et al., 2014); thus they were assumed to be strongly correlated (r = +0.5) (Laurenson et al., 2012). Due to the nature of the defined relationships for establishment and mortality it was also necessary to assume a weak correlation (r = −0.2) between minimum mortality and maximum establishment (Berk et al., 2016b). All other traits were assumed to be uncorrelated (Doeschl-Wilson et al., 2008). Further, random variation in feed intake was included to achieve a phenotypic correlation between food intake and growth rate of approximately 0.8 (Cammack et al., 2005).

### 2.2 Epidemiological module

In the epidemiological module of Berk et al. (2016b), the grazing pasture was defined by the number of hectares and pasture available for grazing (Sibbald et al., 2000), taking into account grass growth and grass consumption on a daily basis. Pasture was assumed to be initially contaminated with overwintered eggs and larvae; subsequent larval contamination of pasture was assumed to arise from eggs excreted by infected calves. The development period from eggs to larvae and the larval mortality were assumed to be temperature-dependent (Stromberg, 1997); the resultant larvae on pasture were considered to have an aggregated distribution. Calves were assumed to graze randomly across the pasture (Laurenson et al., 2011) and consume larvae, removing them from pasture, thus completing the parasitic lifecycle.

### 2.3 Parasite anthelmintic resistance

The mechanism for the development of anthelmintic resistance by *O. ostertagi* to a wide spectrum of anthelmintics is currently not well understood; however there is growing evidence to support a polygenic mechanism (Wolstenholme et al., 2004, Gilleard and Beech, 2007, Prichard, 2007, Yazwinski et al., 2009, Kotze et al., 2014). In the first instance resistance to a single anthelmintic drug, ivermectin, was assumed to be controlled by two genes, each consisting of two alleles. Subsequently, nine possible allele combinations were identified (Barnes et al., 1995). Each allele was assumed to have equal expression within the phenotype (i.e. perfect gene and allele neutrality) hence conveying the same degree of either resistance (R) or susceptibility (S) (Barnes and Dobson, 1990). Ivermectin action was segregated into four key components; a) the degree of dominance of the resistance allele (R), b) drug efficacy against each nematode genotype, c) drug efficacy against each parasitic developmental phase and d) the persistence activity of the drug, which was assumed to be a pharmacokinetic trait of the drug and thus independent of resistance (Smith et al., 1999).

**Fig. 1:**
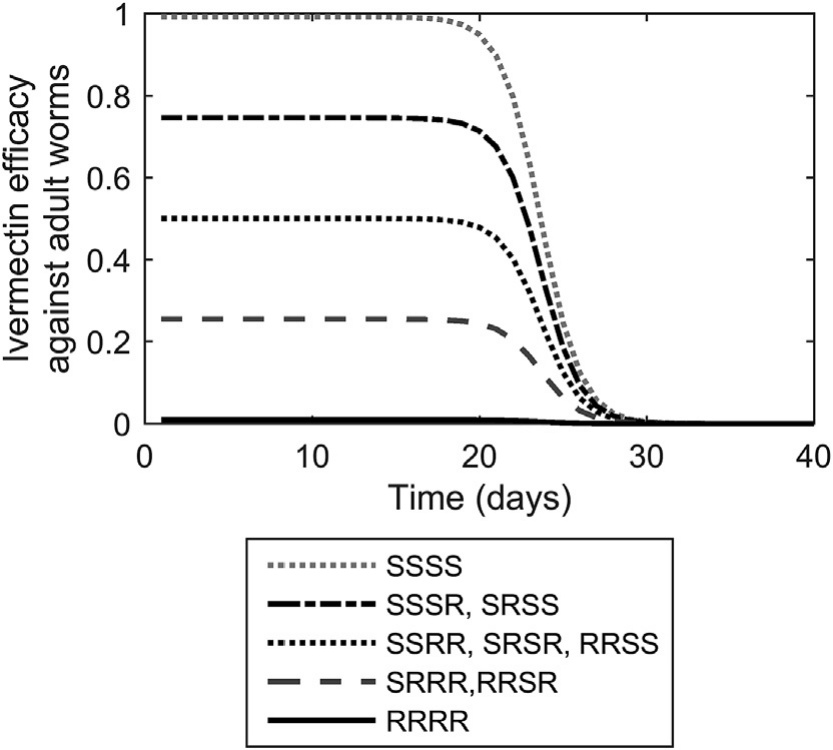
The assumed efficacy, i.e. the mortality success of the drug, over time for which a single treatment with ivermectin is effective against adult *Ostertagia ostertagi*. The efficacy is shown for corresponding worm genotypes with zero, one, two, three and four alleles for resistance; each R allele is assumed to decrease drug efficacy by equal amounts.

The nine possible genotypes constitute 4 different phenotypic expressions; these were assumed to show a graded response from susceptible (S_1_S_1_S_2_S_2_) to resistant (R_1_R_1_R_2_R_2_) (Barnes et al., 1995), dependent on the number of R alleles present as represented in Fig. 1. For example, the genotype combination S_1_S_1_ R_2_R_2_ would be considered to have the same phenotype as S_1_R_1_S_2_ R_2_. Additionally it has been observed that the efficacy of ivermectin is not the same across all stages of development (Eddi et al., 1997, Vercruysse et al., 2000, Yazwinski et al., 2009), hence the efficacy for each stage was defined according to Yazwinski et al. (2009).

Ivermectin is known to display persistent activity of between 1 and 4 weeks against gastrointestinal nematodes in cattle when administered subcutaneously at a rate of 200 μg/kg bodyweight (Armour et al., 1985, Borgsteede and Hendriks, 1986, Williams and Broussard, 1995, Ranjan et al., 1997). This variation in the length of persistent activity can be explained by innate differences in sensitivity amongst various nematode species, environmental factors, such as level of infection (Vercruysse et al., 2000) and within and between differences in pharmacokinetics amongst cattle breeds (Toutain et al., 1997). A curve describing the decay of ivermectin efficacy as a declining sigmoidal function of time was adapted from the equation used by Smith et al. (1999) (equation (1)). The efficacy of a given genotype *x* ($Efficacy_{X}$) at time *t* was defined, whereby efficacy falls between 0 and 1, 0 signifying the drug to have no effect and 1 signifying complete effectiveness.

(1) $$Efficacy_{Xt}=\frac{w_{X}\cdot \exp\left(w_{1}-w_{2}t\right)}{1+\exp\left(w_{1}-w_{2}t\right)}$$

where *t* is time, $w_{1}$ and $w_{2}$ are constants and $w_{X}$ is a parameter that depends on parasite genotype (see below).

Parameters were fitted to published literature to show the expected persistence activity of ivermectin against *O. ostertagi* parasites (Armour et al., 1985, Borgsteede and Hendriks, 1986, Williams and Broussard, 1995, Toutain et al., 1997, Ranjan et al., 1997); as such, *w*_*1*_ = 7.3, *w*_*2*_ = 0.47 and *w*_*X*_ was dependent on the drug efficacy which was defined separately for each genotype according to the number of R alleles present. Drug efficacy against the susceptible genotype was defined according to Yazwinski et al. (2009); however, estimates do not exist for the resistant genotypes. It was therefore necessary to make assumptions about this; it was assumed that drug efficacy against the completely resistant genotype (RRRR) was 0.01 with each R allele assumed to contribute equally to reduction in efficacy (Leathwick et al., 1995, Laurenson et al., 2013). As an example, drug activity against adult worm genotypes is demonstrated in Fig. 1. It was assumed that the initial concentration of anthelmintic increased so rapidly in the host tissues that it was possible to ignore the time taken to reach maximum drug efficacy (Toutain et al., 1997, Lifschitz et al., 2000). Previous versions of the model assumed a persistent activity of 3 weeks against *O. ostertagi*, followed by a decline in efficacy of 0.15 per day for simplicity (Berk et al., 2016b); this was considered a sufficient approximation to the defined curve for the specified treatment.

The resistance genotypes of the initial nematode population on pasture were assumed to arise from random mating, assuming Hardy-Weinberg equilibrium, from an initial frequency of the resistance (allele) assumed to be 0.001 (Barnes and Dobson, 1990). Subsequently, the frequency of R in the worm burden (WB) of each host was used to calculate the frequencies of each genotype in the excreted eggs, again assuming Hardy-Weinberg equilibria. Once the new eggs had hatched and developed into larvae their contribution to the genetic makeup of larvae on pasture was accounted for. It was assumed that all genotypes were equally fit on pasture, such that in the absence of anthelmintic drenching the frequency of R remains the same throughout the simulated grazing season. The total frequency of each genotype in hosts and on pasture was tracked on a daily basis, along with the frequency of R.

### 2.4 Treatment strategies

#### 2.4.1 Timing of treatments

The most appropriate timings for dosing with the antiparasitic drug were determined by simulating a population of untreated calves to predict nematode population (adult worms), pasture contamination (PC) (L_3_/kg DM) and bodyweight gain (kg). It was observed that at approximately 8 weeks post-turnout both parasitic burden and PC began to increase and bodyweight gains were compromised; this coincides with experimental findings in which the majority of calves benefited from treatment at 8 weeks (Höglund et al., 2013, O'Shaughnessy et al., 2015b). In line with O'Shaughnessy et al. (2015a) the simulations support a second treatment at approximately 16 weeks. An 8-week interval between ivermectin treatments in cattle is based on ∼4 weeks of persistent activity against common gastrointestinal nematodes (NOAH, 2015), an average pre-patent period of three weeks and a week of limited exposure to infection (Shaw et al., 1998b). There are no recent studies into the changes in persistence activity due to the build-up of resistance.

#### 2.4.2 Key quantifiable host features

Key quantifiable traits that can be observed non-invasively to provide an indication of the parasitic load (or resulting compromised performance) were identified. Performance can easily be quantified by average daily bodyweight gain (ADG) (kg/d). This was preferable to bodyweight (kg) as variation in initial bodyweights is accounted for; hence any reduction can be attributed to parasitism (Höglund et al., 2009). Direct measures of parasitic load are more complex; the most appropriate and widely used measures were concluded to be faecal egg counts (FEC) (eggs/g) and plasma pepsinogen (international units of tyrosine/litre (IUT/l)), both having their own limitations. Elevated pepsinogen levels occur from around 2–3 weeks as young adult worms emerge from the gastric glands (Jennings et al., 1966, Ritchie et al., 1966). All these traits have shown promising outcomes for the success of TST in practice (Greer et al., 2010, McAnulty et al., 2011, Höglund et al., 2013, O'Shaughnessy et al., 2014a, O'Shaughnessy et al., 2015a, O'Shaughnessy et al., 2015b), therefore relationships for these traits had to be defined within the model.

Pepsinogen concentrations are considered to be a good diagnostic tool for abomasal damage associated with *O. ostertagi* burdens in cattle for the duration of the first grazing season (Charlier et al., 2014); a strong correlation has been observed between adult worm numbers and pepsinogen levels (Anderson et al., 1966, Allen et al., 1970, Baker and Gershwin, 1993, Dorny et al., 1999). Concurrent measurements of WB and pepsinogen were obtained from existing literature (Anderson et al., 1966, Snider et al., 1981, Williams et al., 1987, Baker and Gershwin, 1993, Almería et al., 1996, Szyszka and Kyriazakis, 2013). In the case of Szyszka and Kyriazakis (2013) only pepsinogen was recorded, however as the infections were artificially administered it was possible to replicate the experimental conditions within the model and hence simulate predicted WB associated with elevated pepsinogen levels. The model only considers sub-clinical infections, hence pepsinogen levels above 5 IUT/l were ignored, as these are considered to be clinical (Hilderson et al., 1989, Shaw et al., 1997, Vercruysse and Claerebout, 2001). Pepsinogen levels would be expected to increase as adult worms emerge from gastric glands, causing elevated abomasal pH and leakage due to increased mucosal permeability (Jennings et al., 1966, Allen et al., 1970, Fox et al., 1987); they will continue to increase with increasing WB until a plateau is achieved as no further changes in gastric function occur (Dorny et al., 1999). Consequently, a monomolecular growth function was fitted to the published data for concurrent WB and pepsinogen levels; this equation provided the best fit and mirrored the expected relationship between WB and pepsinogen:

(2) $$Pepsinogen=Pep_{max}-\left(Pep_{max}-Pep_{min}\right)\cdot \text{exp}\left(-C_{p}\cdot Wormburden\right)\;\left(\text{IUT/l}\right)$$

where *Pep*_*max*_ is the maximum pepsinogen for a sub-clinical infection (3.8 IUT/l), *Pep*_*min*_ is the minimum pepsinogen, observed in a healthy calf (0.8 IUT/l) and $C_{p}$ is a rate constant defining the relationship between WB and pepsinogen level (1.67 × 10^−5^) (R = 0.603, RMSE = 0.636). Pepsinogen levels do not provide an exact description of the WB, for this reason random variation in pepsinogen was added and parameterised to mirror a correlation between WB and pepsinogen of approximately 0.7 (Anderson et al., 1966, Allen et al., 1970, Baker and Gershwin, 1993, Dorny et al., 1999).

#### 2.4.3 Targeted selective treatment strategies

The aim of this study was to compare the consequences of control strategies and identify the most effective and sustainable method(s). To provide a baseline for comparison, treatment groups included calves administered no treatment and strategically treated calves with the whole group receiving anthelmintic dosing at the time points of 3, 8 and 13 weeks post-turnout; this has been shown to provide good control of parasitic gastroenteritis in set-stocked, first grazing season calves (Shaw et al., 1998a). Subsequently, a variety of TST strategies were simulated (detailed below) using the aforementioned traits of ADG, FEC and pepsinogen as determinant criteria for treatment. A summary of the different TST strategies investigated is provided in Table 1.

##### 2.4.3.1 TST based on herd percentages

One specification for TST is to dose a fixed percentage of calves selected according to a pre-determined criterion (Laurenson et al., 2013). In order to investigate a range of scenarios, treatments were assumed to occur for 10, 25, 50 and 100% of the host population, as indicated by each of the determinant criteria; 100% signifying whole group targeted treatment. Calves within a population were treated at the specified times, subject to a determinant criterion: for ADG the calves with the lowest gains were preferentially treated; for FEC and pepsinogen the calves with the highest values were preferentially treated. A total of 2 days was allowed for processing and analysis of the samples; ivermectin was then assumed to be administered the following day at 200 μg/kg bodyweight (Höglund et al., 2013, O'Shaughnessy et al., 2014a). An additional comparison group was included whereby calves were selected for treatment at random by generating random pseudo-numbers relating to calf ID numbers; as such, the other determinant criteria were evaluated in relation to this.

##### 2.4.3.2 TST based on threshold values

In contrast to selecting a fixed percentage of the herd for treatment, TST can also be achieved by dosing calves when a determinant criterion reaches a threshold level (Charlier et al., 2014). The same 3 determinant criteria were investigated, with the addition of a group of calves treated according to a combination of FEC and pepsinogen, as this strategy has been investigated in the field (O'Shaughnessy et al., 2014a, O'Shaughnessy et al., 2015a, O'Shaughnessy et al., 2015b). Available literature was used to define threshold values for each determinant criterion. When using ADG as the determinant criterion, calves were treated when individual ADG was inferior to the ADG averaged over the poorest growing 50% of calves in a strategically treated group (3, 8 and 13 weeks) (Höglund et al., 2013). The threshold for FECs was considered to be 80 eggs/g. A trigger of 200 eggs/g has been used previously, however this was defined for mixed infections (O'Shaughnessy et al., 2014a). Although seasonal variation in egg ratios is observed in temperate regions (Dorny et al., 1988, Vercruysse et al., 1988, Verschave et al., 2014), for simplicity it was assumed that an average proportion of 0.4 was *O. ostertagi* eggs (Dorny et al., 1988, Vercruysse et al., 1988, Hilderson et al., 1990, Ploeger and Kloosterman, 1993, Almería et al., 1996, Areskog et al., 2013, Verschave et al., 2015). The threshold for pepsinogen levels was assumed to be 2 IUT/l and therefore the final group involved treating calves when both FECs greater than 80 eggs/g and pepsinogen levels greater than 2 IUT/l were attained by an individual. For all determinant criteria, trait measurements were assumed to be taken every 3 weeks starting from 8 weeks post-turnout (Greer et al., 2010, Höglund et al., 2013, O'Shaughnessy et al., 2014a, O'Shaughnessy et al., 2015a, O'Shaughnessy et al., 2015b) and treatment applied to individuals presenting measurements above or below the specified threshold. The reduction in anthelmintic use was calculated as a percentage of the total anthelmintic applications administered in the strategically treated group.

### 2.5 Simulation procedure and outputs

A population of 500 calves was simulated on pasture over their first grazing season for a period of 6 months from weaning. All calves were assumed to be parasitologically naïve prior to turn-out to pasture at a conventional stocking rate of 5 calves/Ha (AHDB, 2013) and an initial PC of 200 L3/kg DM (Larsson et al., 2007). The same population was modelled for all treatment groups. All model simulations were programmed in MATLAB (2015b).

A population of calves was simulated for each of the selected strategies. Outputs were recorded on a daily basis and compared for the following: performance traits (population average of empty body weight (EBW, kg)), parasitological traits (population average of WB and FEC), epidemiological traits (PC (L_3_/kg DM grass)) and anthelmintic resistance traits, such as the frequency of R in the nematode population on pasture and total number of anthelmintics administered over the grazing season.

Each modelled strategy was compared with the untreated group for its effect on average EBW (providing a similar output to carcass weight) and R allele frequency at the end of the first grazing season (Laurenson et al., 2016). The average weight gain benefit arising from treatment (AWGB, kg) was calculated at the end of the first grazing season when animals were taken off pasture and moved indoors, which was defined as housing (*h*):

(3) $$AWGB_{h}=EBW_{TST_{h}}-\;EBW_{C_{h}}\;\left(\text{kg}\right)$$

where $EBW_{TST_{h}}$ is the EBW at the time of housing (*h*) for a group of calves receiving a given TST strategy and $EBW_{C_{h}}$ is the EBW at time of housing for a group of calves left untreated.

Similarly, for each treatment strategy the frequency of R allele was compared with the untreated control group to determine the impact upon anthelmintic resistance. The increase in R allele frequency ($IRAF_{h}$) from turnout to the end of the grazing season was calculated at housing:

(4) $$IRAF_{h}=RAF_{TST_{h}}-\;RAF_{C_{h}}$$

where $RAF_{TST_{h}}$ is the frequency of the R allele on pasture at time of housing (*h*) for a group of calves receiving a given TST strategy and $RAF_{C_{h}}$ is the frequency of the R allele on pasture at time of housing for a group of calves left untreated.

In order to evaluate each of the simulated strategies, the ‘benefit per R’ (BPR) was calculated to account for production benefits and the impact on anthelmintic resistance such that equal weighting was given to both traits. BPR at time of housing was calculated according to Laurenson et al. (2016) as follows:

(5) $$BPR_{h}=\frac{AWGB_{h}}{IRAF_{h}}\;\left(\text{kg/R}\right)$$

As such, the best strategy will be the one displaying the highest value for BRP.

To make a comparison of the benefit gained from treating a percentage of calves according to each determinant criteria relative to random selection, a number of outputs were assessed in terms of their final predicted values at the end of the grazing season (day 180); these were: A) cumulative faecal egg counts as a measure of parasitism; B) relative reductions in EBW gain as a measure of performance; C) frequency of R on pasture as a measure of resistance and D) BPR value. A two-tailed Z test was carried out to assess the statistical significance of treatments according to each determinant criterion, with the exception of relative reductions in EBW gain which were assessed using the Mann-Whitney *U* test due to the skewed data distribution. For outputs related to resistance (frequency of R on pasture and BPR) the output was a single measure for the complete pasture and therefore variation was estimated by simulating 10 populations for each treatment group. In each simulation, all stochastic parameters describing individuals and their environment were assigned based on a different unique sequence of computer-generated random numbers. The statistical tests revealed whether treatments according to determinant criteria produced outputs different from what might be obtained by random selection. All statistical comparisons were carried out to the 95% confidence level. Additionally, the model recorded which individuals were treated at each assessment, from this the number of treatments shared between groups treated according to different determinant criteria was calculated along with the number of repeat treatments made within each treatment group, i.e. percentage of the individuals receiving treatment at the first assessment to also receive treatment at the second assessment. A comparison of traits used for the threshold treatments was made for BPR using the same methods, there was no standard control to compare all treatments to and therefore they were compared with one another.

## 3 Results

### 3.1 TST based on herd percentages

#### 3.1.1 Comparison of treatment percentages

**Fig. 2:**
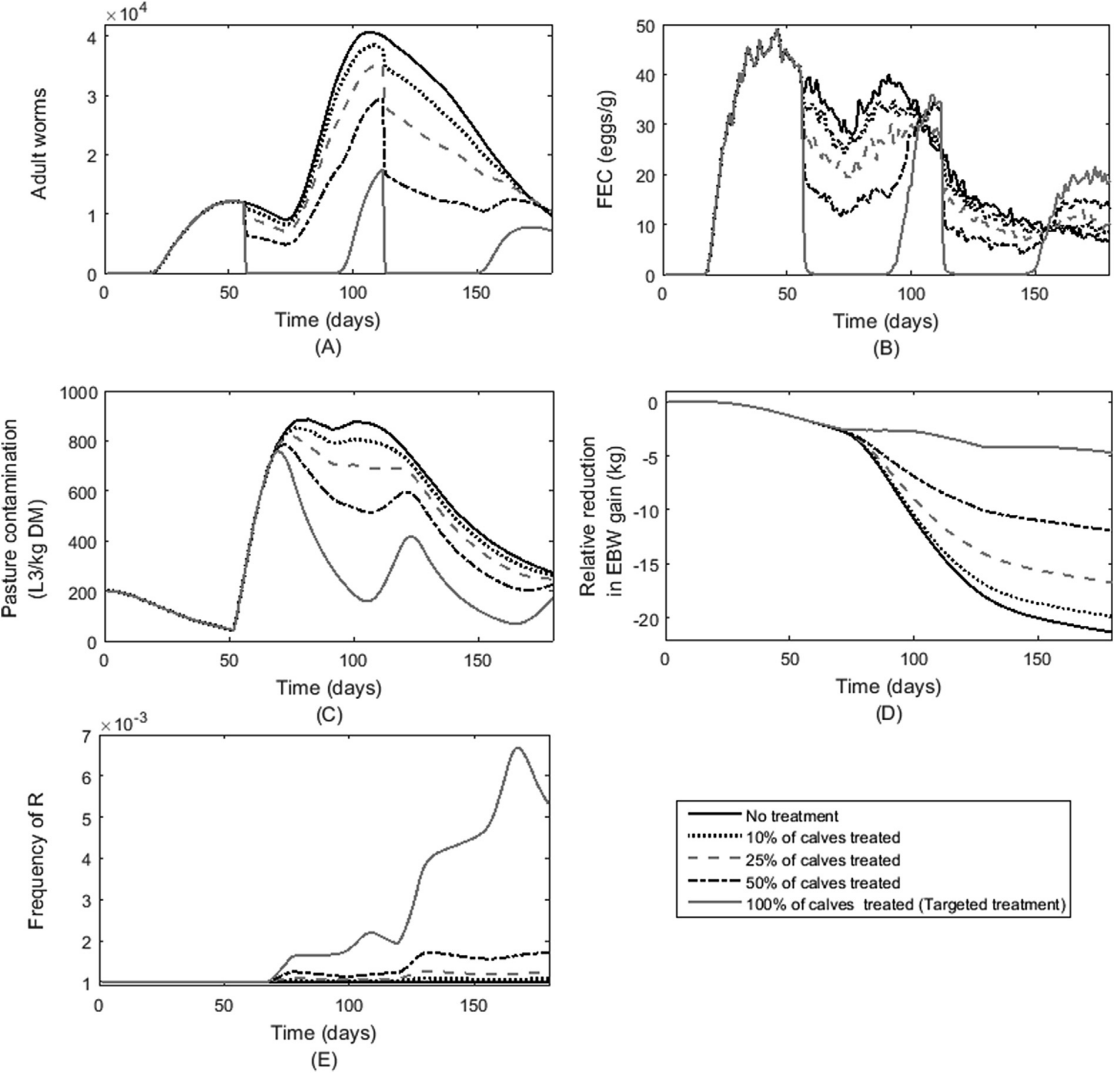
Predictions for groups of calves either left untreated or treated at weeks 8 and 16 according to lowest average daily bodyweight gain (ADG, kg/d) when a percentage of 0, 10, 25, 50 and 100% of a herd of 500 calves grazing on pasture initially contaminated with 200L_3_/kg DM grass were treated with ivermectin; the population averages are presented for outputs of A) worm burden; B) faecal egg output (FEC) (eggs/g); C) pasture contamination (L_3_/kg DM grass); D) relative reduction in empty bodyweight gain relative to a non-parasitised population (kg) and E) the frequency of resistant parasite genotype R on pasture.

The impact of different percentages of treated calves was investigated for determinant criteria of ADG, FEC and pepsinogen. The pattern of outcomes for different percentages of the population treated was similar for all determinant criteria and for this reason the outputs for the determinant criterion ADG are shown on Fig. 2. The impact of treatments on the parasitological output of average WB (Fig. 2A) over one grazing season showed a reduction in peak WB, remaining below that of the untreated group throughout the grazing season. The larger the percentage of calves treated the lower the average WB. This pattern was reflected in the average FEC (Fig. 2B); average FEC was reduced from the first anthelmintic treatment on 56 day post-turnout (dpt) until approximately 105 dpt, when all groups showed an increase in FEC to values equal to or greater than those of an untreated group of calves. The effects were more pronounced when a greater percentage of calves were treated. Following the second anthelmintic treatment, FECs were again reduced relative to the percentage treated; at approximately 155 dpt all treated groups showed an increase to levels above the untreated group. The observed increase was larger when a greater percentage of calves were treated, with the 100% treated group showing the largest final FEC.

Pasture contamination (PC) expressed as L3/kg grass DM (Fig. 2C) was reduced by the treatments relative to the untreated herd, the extent of the reduction was higher the greater the percentage of calves treated. For the group treated at 100%, PC continued to rise for 2–3 weeks following the first treatment due to developing eggs already present on pasture pre-treatment. A subsequent trough in PC was observed. The final PC was approximately the same for all treated and untreated groups. As a result of lower WB and PC prompted by anthelmintic treatments the impacts of parasitism on the relative reduction in EBW gain (compared with a healthy control population) was less for the groups with the highest percentage of calves treated for any of the determinant criteria (Fig. 2D).

Predictably, the treatments most successful at reducing parasitological burdens and reductions in weight gain were also most likely to result in a high frequency of resistant (R) alleles in the nematode population at pasture. Fig. 2E shows the change in frequency of R allele on pasture; as would be expected the larger the percentage of treated calves the greater the increase in R allele frequency, with disproportionally large increases observed when the percentage treated was increased. For example, the increase in frequency of R was 0.0007 and 0.0043 when 50 and 100% of the population were treated. In all cases the frequency of R increased following each anthelmintic treatment. Increasing the percentage of the population treated increased the impact upon the R allele frequency. As such, the pattern predicted for the largest treatment percentage of 100% (whole-herd treatment) was the most exaggerated as a consequence of a reduction in S alleles in eggs deposited onto pasture and a reduction in PC. Following this initial increase, the R allele frequency continued to vary as a consequence of the impact of treatment upon PC coupled with the continued persistent activity of ivermectin. This effect was most notable as a secondary peak in R allele frequency on pasture, prior to the impact of the second anthelmintic, for the whole-herd treatment group. This secondary peak in R allele frequency decreased around 115 dpt reflecting the increase in PC as the persistent effect of ivermectin reduced.

#### 3.1.2 Comparison between determinant criteria

**Fig. 3:**
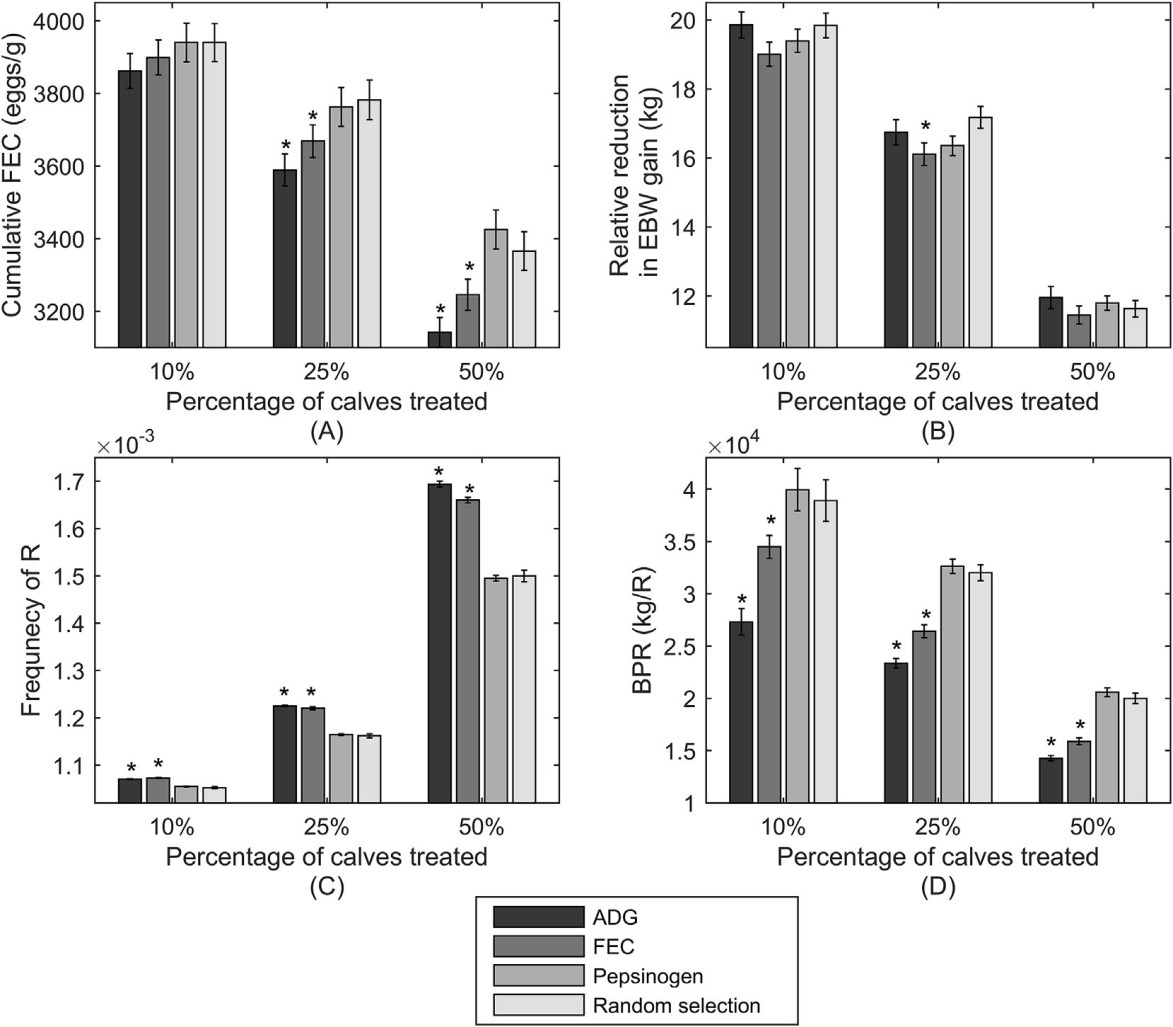
End of season (day 180) predictions for: A) cumulative faecal egg count (eggs/g), B) relative reduction in empty bodyweight gains (kg) in comparison to a non-parasitised population, C) frequency of R on pasture, and D) benefit per R (BPR) representing the benefit in empty bodyweight gain (kg) per change in frequency of R; for 500 calves grazing on pasture initially contaminated with 200L_3_/kg DM grass. Anthelmintic treatment was administered at weeks 8 and 16 to either 10, 25 or 50% of the population according to lowest average daily bodyweight gain (ADG, kg/d), highest faecal egg count (FEC, eggs/g), highest plasma pepsinogen (IUT/I) or selected at random. Predictions for frequency of R on pasture and benefit per R (BPR) are provided as an average of ten simulations. Statistical indications are provided for each treatment group in comparison to those selected for treatment at random.

Fig. 3 provides a comparison of population averages for cumulative FEC, final relative reduction in EBW (in comparison to uninfected controls), final frequency of R on pasture and BPR value for groups of calves drenched at different percentages according to the different determinant criterion traits of ADG, FEC, pepsinogen or random selection. The optimal determinant criterion would be the one that offers a small change in the frequency of R whilst preventing extreme reductions in EBW gains. A statistical comparison of the benefits to cumulative FEC, reduction in final EBW gain, final frequency of R on pasture and BPR of treating according to each determinant criterion was made in relation to treating according to random selection.

Fig. 3A shows the population average and standard error of cumulative FEC, which was used as an indicator of parasitism. Treating calves according to pepsinogen levels showed similar effects to random selection, whereas treatment according to the determinant criteria of FEC or ADG was more effective at reducing FEC, with ADG being predicted to have the greatest improvement over random selection, significant for groups where 25% and 50% of calves treated. The differences between determinant criteria increased with increasing percentages of treated calves, with the FEC treated group also showing significant improvements over the random selection group when 25% and 50% of the population was treated.

Fig. 3B shows the consequences of parasitism on performance; all groups showed similar reductions in final EBW gain. Groups treated according to FEC yielded marginally greater improvements in EBW gain (i.e. smallest relative reduction in EBW gain in comparison to a non-parasitised group), with the difference being significant when 25% of the population was treated. In contrast, groups treated according to ADG showed the least improvement (i.e. largest relative reduction in EBW gain in comparison to a non-parasitised group), whilst being accompanied by the largest range of values within the population. In contrast to cumulative FEC outcomes, the final frequency of R shown in Fig. 3C was highest for groups treated according to FEC and ADG with a significant increase observed relative to calves treated according to random selection for all treatment percentages, whereas there was no significant difference between calves treated according pepsinogen and random selection. Again, this effect was clearer for greater percentages of treated calves. This was conveyed in the BPR values (Fig. 3D): calves treated according to ADG and FEC showed a significantly lower value than predicted for random selection, whereas when treated according to pepsinogen there was no statistical difference. The largest differences between determinant criteria were observed when a smaller percentage of calves were treated, along with the largest variation between populations. When 100% of the herd was treated there was no difference between determinant criteria and therefore it was not possible to conduct a statistical comparison, however it should be noted the average BPR value was 4012 (181) which is notably lower than the value observed for any of the other treatment percentages described.

Treatment strategies were further compared by examining the individuals selected at each treatment stage. Table 2 gives the percentage of total treatments that were shared between populations treated according to different determinant criteria. As can be seen, treatment according to ADG or FEC shared more individual treatments than would be expected by random probability whereas ADG and pepsinogen shared fewer. It was also possible to examine whether individuals treated on the first occasion are more or less likely to be selected on the second occasion; this statistic is also shown in Table 2. Both ADG and FEC showed a greater number of repeat treatments than would be expected at random with ADG showing the largest number of repeat treatments. Conversely, groups treated according to pepsinogen showed fewer repeat treatments than would be expected at random.

### 3.2 TST based on threshold values

**Fig. 4:**
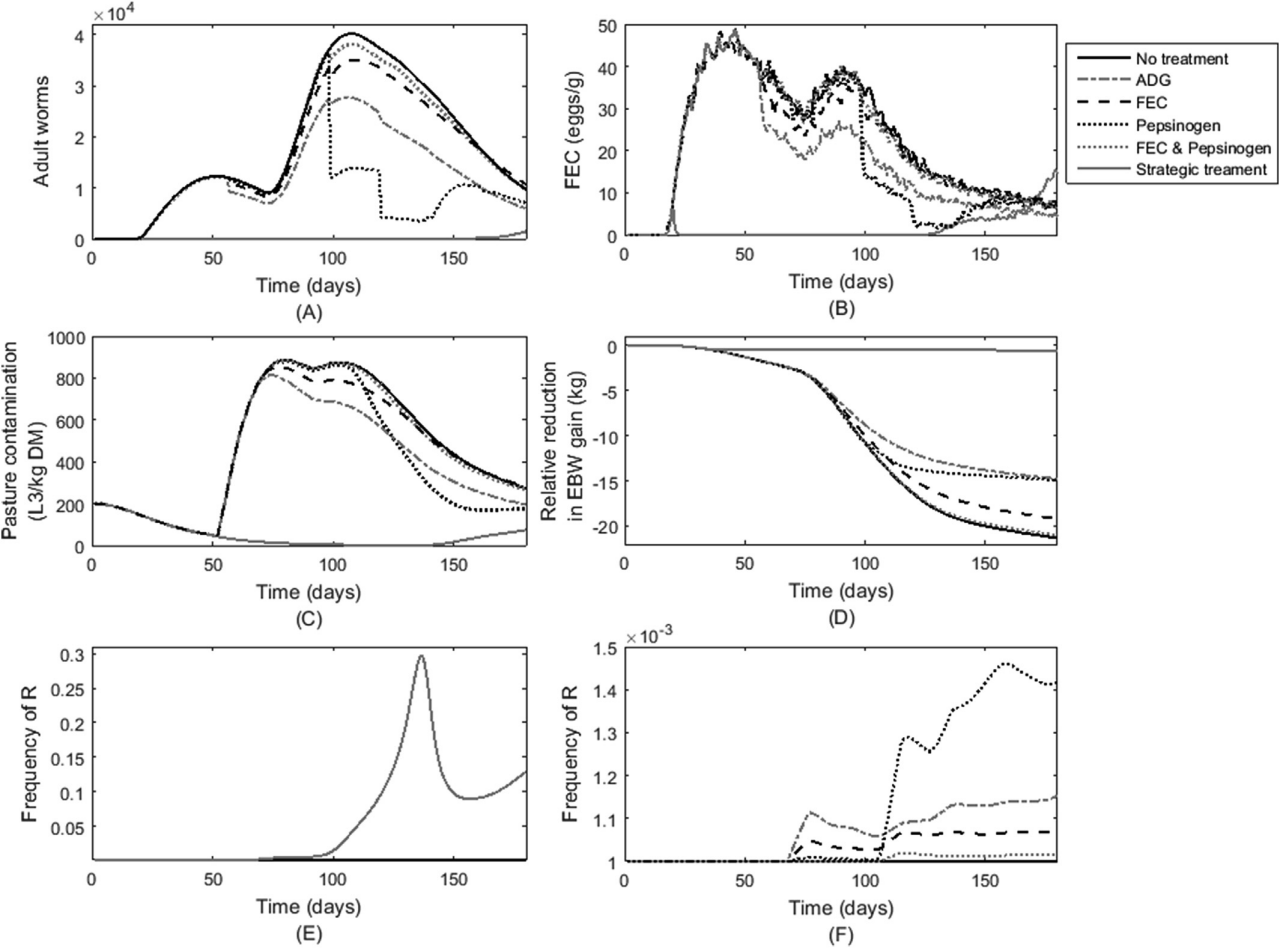
Predictions for groups of calves either left untreated, strategically treated with ivermectin at 3, 8 and 13 weeks post-turnout, or treated with ivermectin according to threshold values for different determinant criteria of average daily bodyweight gain (ADG, kg/d), faecal egg count (FEC, eggs/g), plasma pepsinogen (IUT/l) or the combination of values for FEC and plasma pepsinogen; were made for a herd of 500 calves grazing on pasture initially contaminated with 200L_3_/kg DM grass. The population averages are presented for outputs of A) worm burden; B) FEC (eggs/g); C) pasture contamination (L_3_/kg DM grass); D) relative reduction in empty bodyweight gain relative to a non-parasitised population (kg); E) the frequency of R on pasture for the strategically treated group and F) the frequency of R on pasture for the remaining strategies.

The impact of defining a threshold level for treatment for the different determinant criteria of ADG, FEC, pepsinogen and the combination of FEC and pepsinogen was assessed in terms of the parasitological outputs of WB (Fig. 4A) and FEC (Fig. 4B). Following the first assessment for treatment, all groups showed a lower peak in WB and FEC than that of an untreated group of calves, although the reductions observed were minimal in the group treated according to a combination of FEC and pepsinogen. The largest reductions in WB, and consequently FEC, were observed when the determinant criterion was pepsinogen; this was then followed by groups where the determinant criterion was ADG and finally FEC. Reductions in WB and FEC started earlier when ADG and FEC were used as determinant criteria, compared with the other groups with notable sudden decreases in WB for the pepsinogen group from 98 dpt. For the remaining determinant criteria the decline in WB and FEC was smoother across the grazing period. The strategically treated group in which the whole-herd treatments were applied at 3, 8 and 13 weeks post-turnout showed very low burdens for the duration of the season, with a clear increase observed at the end of the season (from 130 dpt).

As per parasitological traits there was a reduction in peak PC relative to an untreated control for all treatment groups (Fig. 4C); again the decrease predicted for combined FEC and pepsinogen showed minimal reductions. The PC predictions for determinant criteria largely mirrored the predictions in WB and FEC with the reduction occurring more rapidly when pepsinogen was used as the determinant criterion. All treatment groups showed an improvement upon the untreated group for relative reduction in body weight gain in comparison to a non-parasitised population (Fig. 4D). Consistent with reduced parasitological burdens and PC, the groups treated strategically showed EBW close to that expected of a healthy (non-parasitised) calf. The groups treated according to pepsinogen and ADG showed the least reductions in EBW relative to a healthy (non-parasitised) population of calves, followed by FEC, and then the combination of FEC and pepsinogen which showed minimal improvements compared with an untreated groups of calves.

However, upon comparing the frequency of R in the group administered strategic treatment (whole-herd treated at 3, 8 and 13 weeks post-turnout) an increase in the frequency of R (Fig. 4E) compared with all other strategies was evident, with large increases observed up until approximately 135 dpt, coinciding with the increase in eggs excreted to pasture. For this reason outputs for this treatment are shown separately. Fig. 4F shows the frequency of R for the different TST groups. The group treated according to the determinant criterion of pepsinogen alone was seen to give the largest increase in R, followed by ADG and FEC treated groups both of which showed an increase in frequency less than half that of the pepsinogen group. In agreement with other outputs, the group treated according to both FEC and pepsinogen showed minimal changes in the frequency of R.

**Fig. 5:**
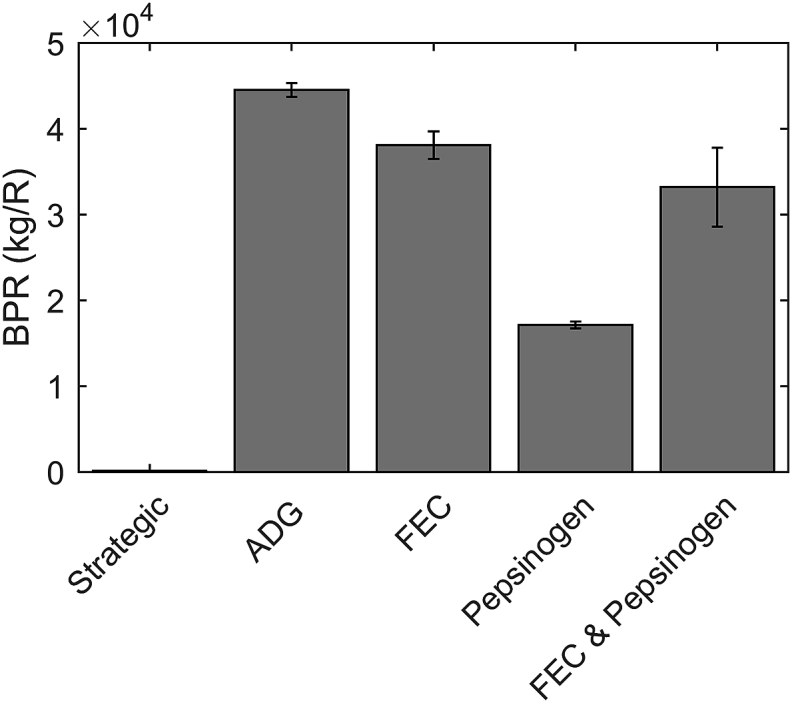
Benefit per R (BPR) simulated at the end of the grazing season (day 180) on a population basis for each of the simulated control strategies; BPR represents the benefit in empty bodyweight gain (kg) per change in frequency of R on pasture, so the higher the value the more beneficial the strategy is perceived to be. Ten discrete populations of calves were simulated on pasture initially contaminated with 200L_3_/kg DM grass for calves treated strategically with ivermectin at 3, 8 and 13 weeks post-turnout or according to threshold values of average daily bodyweight gain (ADG, kg/d), faecal egg count (FEC, eggs/g), pepsinogen or a combination of FEC and pepsinogen. Statistical comparisons were made between groups and are reported within the text.

Fig. 5 represents the BPR values for each of the strategies; the highest value and therefore most beneficial was attributed to the group treated according to ADG. FEC was the next best strategy, closely followed by those treated according a combination of FEC and pepsinogen, then pepsinogen alone. Strategically treated groups were predicted to have a dramatically lower BPR value. The difference between each treatment group was observed to be substantial in all cases. Additionally, the reductions in anthelmintic applications for each strategy compared with strategic treatment were calculated and revealed that the combination of FEC and pepsinogen showed reductions of 98.3%, closely followed by treatment according to FEC for which a 93.4% reduction was observed. Considerably more treatments were applied for ADG and pepsinogen treated groups with reductions of 47.0% and 68.4% respectively.

## 4 Discussion

With the emergence of anthelmintic resistance in GI parasites of cattle (Edmonds et al., 2010, Sutherland and Leathwick, 2011, O'Shaughnessy et al., 2014b, Rose et al., 2015) there have been attempts towards developing TST strategies for cattle. This is important, as although resistance has been slow to develop amongst cattle parasites, it appears that multi-drug resistance for multiple parasite species is developing more rapidly than expected (Sutherland and Leathwick, 2011). There are a number of challenges to address when developing and assessing such strategies. The first is the basis upon which these strategies are developed. Secondly, it is difficult to make direct comparisons on the effectiveness of such strategies through field studies due to confounding variables, such as climatic conditions or management techniques (O'Shaughnessy et al., 2015a). These will have consequences on the underlying infection levels and subsequently affect the perceived success of any treatment strategy. It is therefore unclear from the literature as to which strategy might be most beneficial in treating the effects of parasitism whilst delaying the development of resistance. Finally, in practice it is difficult to assess the development of resistance, especially over a short time-scale (Besier, 2012, Sutherland and Bullen, 2014), which is usually the case with experimentation. Currently faecal egg count reduction tests (FECRT) are used to assess this, however this technique has only been validated for sheep and not cattle nematodes (Sutherland and Bullen, 2014). Compared with sheep nematodes, *O. ostertagi* tends to show less aggregation between hosts, excrete fewer eggs (Demeler et al., 2010, El-abdellati et al., 2010, Yazwinski et al., 2013) and FEC are generally less reflective of WB as a result of density-dependent effects on parasite fecundity (Michel et al., 1978, Smith et al., 1987). As a result, the limited numbers of studies conducted on cattle TST have focused on performance and total number of anthelmintic applications. In this paper, the relative success of different TST applied here was evaluated on the basis of BPR, the ratio of average benefit in weight gain to change in frequency of R (relative to an untreated population).

With these difficulties in mind we embarked upon further developing a recently published population model to predict the consequences of different TST strategies on cattle and their *O. ostertagi* populations (Berk et al., 2016b). We were particularly interested in the consequences of: 1) the most appropriate determinant criteria for treatment selection and 2) the method of selecting animals for treatment, the contrast being treating a fixed percentage of the population with the lowest (or highest) trait values versus treating individuals who exceed (or are below) a given trait threshold for treatment. As the model was population-based, it allowed us to trace individual animals within a group and select individuals on the basis of the different methods. The model was applied to first season grazing calves infected with *O. ostertagi*, the most important parasite affecting health and productivity in temperate climates. Strategies were selected on the basis of literature; however different methods of defining threshold triggers have been proposed, in particular for ADG. Höglund et al. (2009) suggested that an ADG below 0.75 kg/d would provide a good trigger threshold for treatment; however a set value does not account for the sigmoidal nature of growth or indeed for variability in intrinsic growth between and within genotypes. For example, healthy calves that are close to their maximal weights and hence show slower growth would be considered to require treatment. However, the risk of this misinterpretation may not be a major concern for the time interval considers, as calves were probably in the linear growth phase. Greer et al. (2010) and McAnulty et al. (2011) proposed determining the expected ADG for individuals at any given time point dependent on individual calf bodyweight measured at the previous time point. Although a significant improvement to the previous method, there are also problems associated with this, which arise from the natural uncertainty associated with a single body weight measurement. The selected method, based on the mean ADG of the poorest 50% of calves in a strategically treated population, was assumed to provide conservative estimates of the expected ADG in a healthy population hence indicating individuals who showed an ADG below this expectation.

As expected, all of the simulated TST regimens improved weight gains and reduced most (but not all) measures of parasitism compared with an untreated herd. The methods used for selection and their determinant criteria predicted different outcomes and therefore each is addressed separately.

### 4.1 TST based on herd percentages

A comparison of the impact (upon various traits) of treating 10, 25, 50 or 100% of calves according to the determinant trait ADG is provided in Fig. 2. Upon treating 100% of the population individuals experienced a temporary elimination of parasitological burden, but later displayed a rebound effect – a steep rise in infection. This rebound effect was a consequence of a reduced rate of immune acquisition due to a reduction in antigen exposure in the treated individuals. Results for partial treatment of the herd were essentially weighted averages of the untreated and treated predictions. As a consequence of reduced WBs the treated individuals experienced reduced protein loss and increased feed intake (due to reduced immune acquisition), hence resulting in greater average EBWs when a greater percentage of calves were treated. Larger calves with larger feed intake requirements consumed higher quantities of grass, and therefore a greater proportion of the larvae on pasture. Consequently, large numbers of susceptible larvae were removed from pasture and killed by anthelmintic activity, whereas resistant larvae removed from pasture survived within the host to produce eggs. This selective process tended to reduce PC but enriched the fraction of resistant eggs excreted to pasture. Therefore the frequency of R alleles increased as a greater percentage of calves was selected for anthelmintic treatment. Overall it was predicted that treating fewer calves provided the greatest overall benefit as reflected in BPR (Fig. 3). This finding is similar to what has been found when modelling similar TST for lambs (Gaba et al., 2010, Laurenson et al., 2013).

Selection for treatment according to each determinant criterion was compared with random selection on the basis of cumulative FEC, reduction in EBW (relative to non-parasitised group), frequency of R on pasture and BPR (Fig. 3). Determinant criteria of ADG and FEC resulted in reduced cumulative FEC compared with random selection, either through a direct effect on eggs or via the impact of calf size on volume of faeces produced (hence concentration of eggs in faeces). Little absolute difference in reduction in EBW gain (relative to non-parasitised group) was observed between different determinant criteria; however ADG resulted in the largest average reduction in EBW gain. Dissection of the model components revealed this to be a result of varied intrinsic growth rates within the population; a portion of those selected for treatment on the basis of low ADG were intrinsically slow growers and not impeded by parasitism. Additionally a portion of the calves experiencing large reductions in ADG did not receive treatment as they were intrinsically fast growers and their ADG did not fall below that of non-parasitised intrinsically slow growers. Although determinant criteria of ADG and FEC resulted in treatment to many of the same individuals, FEC showed the greatest improvements in EBW. This was due to treatment of intrinsically fast growers impeded by parasitism and a lack of treatments to intrinsically slow growers showing few signs of parasitism. Unlike EBW the determinant criteria substantially impacted on the frequency of R, and therefore BPR. The most efficient strategy was to treat calves with the greatest WB, which suffer the greatest parasite-related loss of productivity, whilst due to density-dependent effects and immune response are contributing less to the aggregate herd excretion of eggs (Michel et al., 1978, Smith et al., 1987). Calves with lower WB may nevertheless have high FEC, and it is advantageous to allow them to continue producing susceptible eggs while their performance is not as severely affected by WB. According to this rationale, pepsinogen selection was the best method to identify the optimal treatment group, whereas ADG and FEC tend to exclude optimal candidates: ADG by selecting intrinsic slow-growers with low WB, and FEC by selecting low to moderately infected calves showing high FECs.

Interactions between the percentage of calves treated and the determinant criteria used for selection were predicted for BPR (Fig. 3). The largest difference in BPR value between determinant criteria was observed when a smaller percentage of calves were treated. For all determinant criteria, treating 10% of the population resulted in the largest variation in BPR values across different calf populations. This implies a greater range of possible outcomes associated with treating fewer calves. Interactions were not observed between treatment percentage and determinant criteria, simulations suggest that these selection criteria of FEC and ADG are counter-productive compared with random or pepsinogen based selection because of their more detrimental effect on refugia for reasons discussed above.

### 4.2 TST based on threshold values

TST based on threshold triggers appeared to show the reverse pattern in terms of the most beneficial determinant criterion compared with treating a fixed percentage of the population (Fig. 5). Treating calves according to thresholds for ADG showed by far the greatest benefit; this was followed by FEC, combined FEC and pepsinogen, and pepsinogen alone. This pattern can be explained by the observations of Fig. 4. Although the modelled treatment for selection according to ADG required the highest number of treatments, the development of resistance remained low. This is explained by a combination of factors: first, the tendency for this method to select calves with an intrinsically slow growth genotype that do not necessarily have high WB. Second, the method does not select tolerant individuals (i.e. individuals in which infection is not limited but negative fitness consequences are offset), experiencing large WBs without showing clear signs of poor performance due to parasitism. The former resulted in only small numbers of resistant alleles contributing to pasture, whereas the latter allowed large numbers of susceptible eggs contributing to pasture.

Treatment according to FEC had similar effects on PC. Simulations showed FEC was highest early in the grazing season, meaning that selection according to FEC resulted in the majority of treatments administered early in the season, preventing the build-up of PC. Conversely, WBs began to rise towards the latter stages of the grazing season in tandem with the expected mid-season rise in PC, causing elevated pepsinogen levels. This resulted in large numbers of treatments administered in unison, therefore causing sudden reductions in WB and PC. Although the EBW recovered, this had significant implications for the frequency of R on pasture. Due to a lack of correlation between WBs (represented here by pepsinogen levels) and FEC there were very few individuals selected for treatment based on the combined criteria of pepsinogen and FEC. However, in this case, greater variation was observed in the BPR between simulated populations than for other determinant criteria.

### 4.3 Comparison of strategies

Upon assessing the best determinant criterion for the two described methods of selection for treatment contrasting patterns were observed. ADG was the best determinant criterion for treating individuals who cross a given threshold for treatment, in accordance with previous work on sheep (Cabaret et al., 2006, Greer et al., 2009, Chylinski et al., 2015). However, ADG was the worst determinant criterion when treating a fixed percentage of the population, in accordance with Laurenson et al. (2013). This paradoxical difference between methods can be explained by the frequency and timing of treatment assessments. When treating calves according to threshold triggers more frequent assessments were made, ADG was a good early indicator of infection and hence by assessing individuals more frequently infection can be caught in the early stages preventing further reductions in ADG or the accumulation of PC. Only two assessments were made when treating fixed percentages of the population; by the second assessment treating calves that displayed the largest reductions in ADG had in general developed a strong immunity, implying little benefit was gained from treatment. Alternatively pepsinogen was the best criterion when treating a fixed percentage of calves, but the worst when treating individuals according to a threshold trigger. Pepsinogen relates closely to WB and abomasal damage providing a good indicator of individuals that are heavily parasitised and display a lack of immunity, and would therefore benefit from treatment (Jennings et al., 1966, Armour and Bruce, 1974, Armour et al., 1979). However, this made pepsinogen a poor indicator when treating according to threshold triggers, being less effective than other determinant criteria at preventing a build-up of PC.

### 4.4 Qualitative validation

Where possible, comparisons were made between model predictions and reported experimental studies. Threshold trigger values for the determinant criteria of ADG and combined FEC and pepsinogen have been tested experimentally (Greer et al., 2010, McAnulty et al., 2011, Höglund et al., 2013, O'Shaughnessy et al., 2014a, O'Shaughnessy et al., 2015a, O'Shaughnessy et al., 2015b). The model predicted treating calves according to threshold triggers for ADG to be the most beneficial strategy, in agreement with Höglund et al. (2009) who conducted a retrospective study on the feasibility of different TST determinant criteria and concluded ADG to be the most promising. In subsequent studies conducted to corroborate this prediction, Greer et al. (2010) made comparisons of two farms of dairy calves treated according to threshold triggers of ADG versus calves receiving routine treatment, with assessments made at monthly intervals. For groups treated according to TST, an average of 0.83 and 1.76 anthelmintic treatments per calf were required for the two farms respectively, representing an 84% and 65% reduction in anthelmintic usage compared to the control group. On both farms the TST groups showed larger within-group variations in bodyweight along with a reduction in ADG of 6% and 4% comparative to the control group routinely treated at monthly intervals. These observations relate well to model predictions: the simulated TST using threshold triggers of ADG required 1.72 treatments per calf and showed a 5% reduction in ADG relative to a non-parasitised calf. To make these comparisons on reduction in ADG it was necessary to assume that the experimental control group (given routine monthly treatment) showed similar ADG to what would be expected of a healthy calf. The method of Greer et al. (2010) was repeated by McAnulty et al. (2011) for two herds. Comparable to Greer et al. (2010) the first herd required 1.4 treatments per calf resulting in a 74% reduction in anthelmintic usage and a 5% reduction in ADG relative to the control group of calves (given routine monthly treatment), supporting model outputs. However, the outcomes on the second herd was less agreeable with model predictions; 3.7 treatments were required per calf representing a 47% reduction in anthelmintic usage and reductions in ADG of 2% relative to the control group were achieved, emphasising the difficulty of making quantitative comparisons even when the same strategy is applied.

Further studies using ADG as a threshold trigger have been conducted for beef cattle. Höglund et al. (2013) compared first grazing season bull calves subject to different treatment strategies. Calves were left untreated, routinely treated every 4 weeks, or treated by TST when the ADG was inferior to the ADG averaged over the poorest growing 50% of calves in the group receiving routine treatment every 4 weeks. A total of 0.6 treatments per calf were required, a 92% reduction in anthelmintic usage when compared with the control group. In general the experimental TST group showed bodyweight gains intermediate to those of untreated and routinely treated groups, but similar FEC to the untreated group. Similar patterns were also predicted by the model; when compared with untreated calves the TST group showed very similar FECs but an improved ADG, although the simulated reductions in bodyweight gain were not always as extreme as those observed in the experiment.

No studies exist investigating the sole use of FEC or pepsinogen as a trait for TST. Recent studies by O'Shaughnessy et al., 2014a, O'Shaughnessy et al., 2015a, O'Shaughnessy et al., 2015b have looked at implementing TST using combined pepsinogen and FEC thresholds, often with a third condition for treatment based on the presence of lungworm. In all studies a control group treated three times was included for comparison. O'Shaughnessy et al., 2014a, O'Shaughnessy et al., 2015b found that no individuals reached both FEC and pepsinogen levels large enough to trigger threshold treatment. Similar to these studies the model predicted very low numbers of treatments required with 0.05 treatments needed per calf. However, O'Shaughnessy et al. (2015a) found 1.5 treatments were required per calf, a 50% reduction in anthelmintic usage of the control group, although only 0.5 were as a result of *O. ostertagi* markers with the majority due to lungworm. Although the reported studies are in good general agreement, there are many confounding variables and only qualitative comparison can be made. Model predictions are subject to the influence of factors such as climatic conditions, nutrition, management practices, presence of other infectious agents and the level of drug resistance, not all of which are described in the reported studies. For example, the low number of treatments required in the studies by O'Shaughnessy et al., 2014a, O'Shaughnessy et al., 2015b was hypothesised to be a result of the low level PC experienced throughout the field trials. Additionally, many of the control groups used in these studies represented more frequent treatments than would be recommended in practice (Höglund et al., 2013).

### 4.5 Perspectives

The developed model gives a detailed analysis of various control strategies formulated to ensure continued effective control of parasitism in the future, providing valuable insights that were previously absent in literature and considerable support for treating calves according to TST. Support was provided for treating fewer calves to help maintain *refugia* and more strongly for treating calves according to threshold trigger values, in particular for the determinant criterion ADG. Trigger thresholds may be considered more applicable across infection levels. For example, over-treatment of herds exposed to very low levels infections may be reduced. Treating according to ADG is beneficial not only in terms of treatment success, but also for ease of practical implementation. However, the modelled trigger threshold for ADG was calculated based on growth rates of their strategically treated counterparts. In practice a group of strategically treated calves would not be kept to calculate this threshold level. One way of overcoming this is by looking at growth trajectories of individual animals and treat animals that deviate from their own trajectory.

In order for these strategies to be adopted farmers must be convinced of the merits of TST. In practice, the implementation of TST on cattle farms requires further optimisation, cost-benefit analysis, and attention to practical issues related to assessment of individuals for treatment. The most feasible option is treatment according to ADG as measurements are instantaneous with fewer additional diagnostic costs. Although currently weighing scales are expensive, individual weighing is labour intensive and poses risk of injury to both cattle and humans, the rapid advances in precision farming may change all these (Laca, 2009). Nevertheless, convincing farmers to convert to TST strategies may not be straightforward, as has been suggested for sheep, especially because the benefits from reducing the rate of anthelmintic resistance development may not be immediately obvious. The relative advantage, complexity and compatibility of TST strategies are all important factors taken into consideration by the farming industry (for which varied priorities exist) (Woodgate and Love, 2012). Difficulties in quantifying such factors make it challenging for farmers to visualise the problem and subsequent benefits of TST, steps towards quantifying these are essential as change is more likely to be adopted when the problem is obvious (Rogers, 1995). Dealing with this challenge, constitutes a new field of research that requires the collaboration between parasitologists and social scientists.

Our model focused on a first grazing season over 6 months however, many calves are kept for a second grazing season or more. Extension of the model to simulate calves over multiple grazing seasons would provide insights into the implementation of these strategies over a longer period. At the end of the first grazing season treatment strategies will have different effects on factors such as final PC, hypobiosis and immunity (Claerebout et al., 1999). All these have important implications for second grazing season calves in terms of infection dynamics, making this an important issue to address in terms of the sustainability of different control strategies. Additionally, most natural infections are mixed with *Cooperia* sp. which can often be more prevalent, particularly in the early stages in the grazing season. It is difficult to distinguish between species in faecal samples, with large numbers of eggs produced by *Cooperia* worms implying threshold values of FEC may not be representative of *O. ostertagi* burdens. Although there does not appear to be inter-species interactions (Kloosterman et al., 1984, Satrija and Nansen, 1993, Hilderson et al., 1995) there are important consequences for levels of protein loss, hence modification of the model to account for *Cooperia* would prove beneficial in future development.

In the model we developed a relationship between ivermectin activity, an anthelmintic widely used in cattle in the UK (Barton et al., 2006), and different *O. ostertagi* genotypes. This was required to determine the effect of treatment on the frequency of resistance alleles (R) within the nematode population. There is now strong evidence that the mechanism for ivermectin is complex and controlled by many alleles at separate loci (Gilleard, 2006, Prichard, 2007, Kotze et al., 2014). To avoid model complexity anthelmintic resistance was assumed to be conferred by two independent genes. There are many unknown factors influencing the rate of anthelmintic resistance, for example the number of relevant alleles, the relative importance of various alleles (on drug efficacy and persistence activity), level of pre-existing alleles and the relative fitness of alleles on pasture (or within an untreated host), amongst others. Should alterations be made to these parameters it would be expected that the rate at which anthelmintic resistance develops would be affected (Barnes et al., 1995, Leathwick, 2013), although the same general principles and patterns would be expected to apply. For example, little indication exists in the literature as to the fitness of each genotype either on pasture or against anthelmintic treatment. Upon modelling a fitness cost associated with R alleles (either on pasture or within an untreated host) it was observed that the development of resistance was slowed, however the same general patterns were observed. Ultimately, the aim of the model was not to accurately predict the rate at which resistance occurs, but rather to compare the relative effect of a range of control strategies.

In conclusion, we have developed a simulation model that appears to be capable of predicting the consequences of TST on the performance and development of nematode resistance amongst calf populations. We suggest that the utility of the model is such that allows it to be extended to consider other strategies for reduction of the development of resistance, including different parasite species and host genotypes and variation in climatic influences on larval availability and grass growth.

## Figures and Tables

**Table 1:** A summary of the different control targeted selective treatment (TST) strategies investigated for differing methods of selection for treatment; ADG = Average Daily Gain (kg/d); FEC= Faecal Egg Counts (eggs/g).

Determinant criteria	Treating a fixed percentage of the population with the lowest (or highest) trait values	Treating individuals who exceed (or are below) a given trait threshold
ADG	√	√
FEC	√	√
Pepsinogen	√	√
FEC and Pepsinogen	–	√
Random selection	√	–

**Table 2:** A comparison of TST strategies whereby 10, 25 and 50% of calves were treated at 8 and 16 weeks either at random or according to lowest average daily bodyweight gain (ADG, kg/d), highest faecal egg count (FEC, eggs/g) or highest plasma pepsinogen (IUT/I). Values provided represent the percentage reduction in anthelmintic use relative to a population of calves treated strategically at 3, 8 and 13 weeks post-turnout. Additionally, the number of treatments shared between groups treated according to ADG, FEC or pepsinogen are provided. Within each treatment group a record was made of the number of individuals that had been treated at the first assessment that were also treated at the second assessment. The expectation of each occurring at random is provided as a comparison.

	Determinant criteria	Percentage of herd treated		
		10%	25%	50%
% reduction in anthelmintic use^a^	–	93%	83%	67%
% of shared treatments between determinant criteria	Random	10.0%	25.0%	50.0%
	ADG-FEC	20.0%	32.0%	55.6%
	ADG -Pepsinogen	4.0%	16.4%	42.2%
	FEC-Pepsinogen	7.0%	27.6%	48.6%
% of first treated group to be selected for second dose	Random	10.0%	25.0%	50.0%
	ADG	84.0%	87.2%	90.0%
	FEC	26.0%	39.2%	70.0%
	Pepsinogen	2.0%	18.4%	40.4%

a Comparative to strategically treated calves (3, 8 and 13 weeks).
